# Targeting Inflammatory Pathways by Triterpenoids for Prevention and Treatment of Cancer

**DOI:** 10.3390/toxins2102428

**Published:** 2010-10-22

**Authors:** Vivek R. Yadav, Sahdeo Prasad, Bokyung Sung, Ramaswamy Kannappan, Bharat B. Aggarwal

**Affiliations:** Cytokine Research Laboratory, Department of Experimental Therapeutics, The University of Texas M.D. Anderson Cancer Center, Houston 77030, TX, USA; Email: vryadav@mdanderson.org (V.R.Y.)

**Keywords:** triterpenoids, nuclear factor-κB, inflammation, tumor cell proliferation, invasion, angiogenesis, apoptosis

## Abstract

Traditional medicine and diet has served mankind through the ages for prevention and treatment of most chronic diseases. Mounting evidence suggests that chronic inflammation mediates most chronic diseases, including cancer. More than other transcription factors, nuclear factor-kappaB (NF-κB) and STAT3 have emerged as major regulators of inflammation, cellular transformation, and tumor cell survival, proliferation, invasion, angiogenesis, and metastasis. Thus, agents that can inhibit NF-κB and STAT3 activation pathways have the potential to both prevent and treat cancer. In this review, we examine the potential of one group of compounds called triterpenes, derived from traditional medicine and diet for their ability to suppress inflammatory pathways linked to tumorigenesis. These triterpenes include avicins, betulinic acid, boswellic acid, celastrol, diosgenin, madecassic acid, maslinic acid, momordin, saikosaponins, platycodon, pristimerin, ursolic acid, and withanolide. This review thus supports the famous adage of Hippocrates, “Let food be thy medicine and medicine be thy food”.

## 1. Introduction

Natural compounds have been used extensively in the treatment of many diseases and are of interest to researchers both in their natural forms and as templates for synthetic modification. Natural compounds currently used in medicine exhibit a very wide chemical diversity, and together with their analogues and several other natural products, they demonstrate the importance of compounds from natural sources in modern drug discovery efforts. Sample sources and molecular mechanisms are highly important in the development of novel, clinically useful anticancer agents [1]. Interest in natural compounds has grown in recent years because of concerns about drug costs and safety. For example, glioblastoma kills almost everyone who gets it, usually in a little over a year. In effect, the $1.3 billion spent by a pharmaceutical company on a new glioblastoma drug discovery had the limited impact of improving patients’ lives for about one year. This illustrates the need for new sources for drug discovery, and natural sources provide valuable information for research in this area. During the past decade, tremendous progress has been made toward understanding the cellular and molecular mechanisms underlying the process of carcinogenesis, leading to the development of potential cancer prevention options termed chemoprevention [2]. The goal of chemoprevention is to use noncytotoxic natural agents to inhibit or reverse the development and progression of precancerous cells [3].

**Figure 1:**
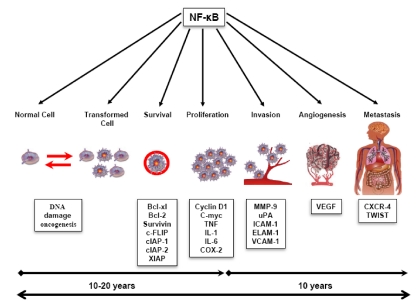
Roles of the NF-κB-mediated inflammatory pathway in cellular transformation and in cancer cell survival, proliferation, invasion, angiogenesis, and metastasis.

Cancer is a complicated disease that may develop in humans over a number of years (Figure 1). Development of a tumor starts with a normal cell that is transformed through the activation of proto‑oncogenes and the suppression of tumor suppressor genes such as *p53*. The transformed cell no longer behaves like a normal cell but begins to exhibit the properties of a cancer cell. Such transformation in the cells makes them self-sufficient in growth signals and resistant to antigrowth signals, resulting in uncontainable proliferation. In addition, these cells are able to avoid apoptosis, resulting in tumor growth. This whole process of transformation may take 10–20 years. The growth of the tumor is aided by angiogenesis, which not only provides nutrition to the tumor but also enables its invasion to surrounding tissues, and its metastasis to distant tissues; the latter is usually lethal.

Inflammation, which occurs as a response to cancer, has two stages, acute and chronic. Acute inflammation, the initial stage of inflammation, represents innate immunity; it is mediated through the activation of the immune system, lasts for a short period and generally is regarded as therapeutic inflammation. If the inflammation persists for a long period of time, however, the second stage, chronic inflammation, sets in [4]. Chronic inflammation has been linked with most chronic illnesses, including cancer, cardiovascular disease, diabetes, obesity, pulmonary disease, and neurologic disease [5], the current review focuses on the role of triterpenoids in targeting inflammatory pathways for prevention and treatment of cancer.

Evidence from tissue culture, animal, and clinical studies suggests that more than 20,000 triterpenoid-rich fruits are found in nature and have the potential ability to limit the development and severity of certain cancers and inflammatory diseases [6]. These triterpenoids, along with their close chemical relatives the steroids, are members of a larger family of related structures called cyclosqualenoids. Triterpenoids, synthesized in many plants by the cyclization of squalene [7], are widely used in Asian medicine. More than 100 prescribed drugs in the United States are obtained from natural sources and represent one fourth of the total drugs used. Apart from these drugs that originate from natural sources, other phytochemicals also serve as potential drugs after structural modification [8].

**Figure 2:**
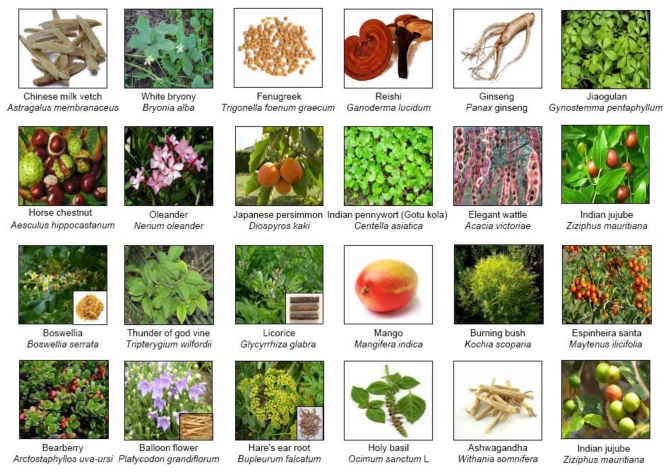
Triterpenoids and their sources.

**Figure 3:**
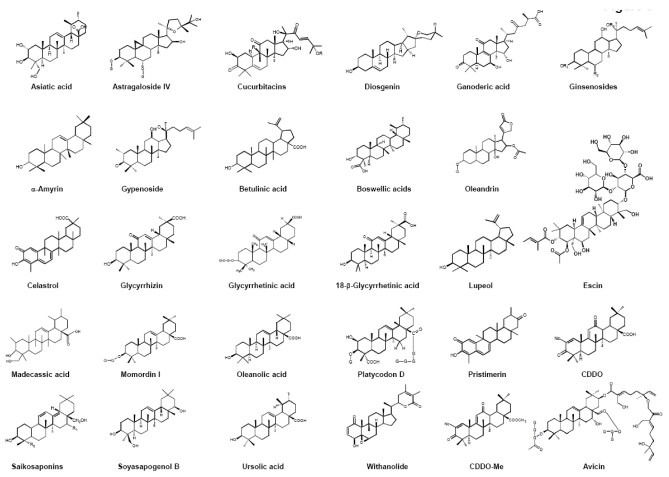
Chemical structures of different types of triterpenoids that inhibit NF-κB.

Scientific studies have shown triterpenoids to be potential anti-inflammatory and anticancer agents. This review covers the anti-inflammatory and anticancer property of triterpenoids originating from plants such as onion, ginseng, brahmi, azuma ichirinsou, shallaki, salai guggal, lei gong teng, licorice, mango, olive, bearberry, Chinese bellflower, sickle-leaf, tulsi, ashwagandha, and others (Figure 2 and Table 1) that target one or more of the various phases of tumorigenesis. As more than 20,000 triterpenoids are available in nature and it is difficult to describe them all, this review summarizes what we know of a few triterpenoids with structural similarity, including avicin, erythrodiol, madecassic acid, maslinic acid, momordin, saikosaponins, 2-cyano-3,12-dioxooleana-1,9(11)-dien-28-oic acid (CDDO) and its methyl ester CDDO-Me, platycodon D, withanolide, diosgenin, betulinic acid, boswellic acids, pristimerin, and celastrol (Figure 3); their active moieties for anti-inflammatory and anticancer activity. 

**Table 1:** Common medicinally active triterpenoid obtained from plants.

Chemical Compound	Common Name	Botanical Name
Tetracyclic triterpenoid		
Astragaloside	Chinese milk vetch	*Astragalus membranaceus*
Cucurbitacin	White bryony	*Bryonia alba*
Diosgenin	Fenugreek	*Trigonella foenum graecum*
Ganoderic acid	Reishi	*Ganoderma lucidum*
Ginsenoside	Ginseng	*Panax ginseng*
Gypenoside	Jiaogulan	*Gynostemma pentaphyllum*
Oleandrin	Oleander	*Nerium oleander*
Pentacyclic triterpenoid		
Amyrin	Japanese persimmon	*Diospyros kaki*
Asiatic acid	Indian pennywort	*Centella asiatica*
Avicin	Elegant wattle	*Acacia victoriae*
Betulinic acid	Indian jujube	*Ziziphus mauritiana*
	Anemone	*Anemone raddeana*
	Club mosses	*Lycopodium cernuum*
	Trumpet satinash	*Syzygium claviflorum*
Boswellic acid	Boswellia,	*Boswellia serrata*
	Frankincense, salai guggal	*Boswellia carteri*
Celastrol	Thunder god vine	*Tripterygium wilfordii*
Escin	Horse chestnut	*Aesculus hippocastanum*
Glycyrrhizin	Licorice	*Glycyrrhiza glabra*
18-β-Glycyrrhetinic acid	Licorice	*Glycyrrhizia glabra*
Lupeol	Mango	*Mangifera indica*
	Three leaved caper	*Crataeva nurvala*
Madecassic acid	Indian pennywort, gotu kola	*Centella asiatica*
Momordin	Burning bush	*Kochia scoparia*
Oleanolic acid	Bearberry	*Arctostaphyllos uva-ursi*
	Heather	*Calluna vulgaris*
	Three leaved caper	*Crataeva nurvala*
	Reishi	*Ganoderma lucidum*
	Chinese elder	*Sambucus chinensis*
	Sodom's apple	*Solanum incanum*
Platycodon D	Balloon flower	*Platycodon grandiflorum*
Pristimerin	Espinheira santa	*Maytenus ilicifolia*
	Pale Bittersweet	*Celastrus hypoleucus*
	Thunder god vine	*Tripterygium wilfordii*
Saikosaponins	Hare's ear root, sickle-leaf	*Bupleurum falcatum L.*
Ursolic acid	Holy basil, tulsi	*Ocimum sanctum L.*
	Thyme	*Thymus vulgaris L.*
	Lavender	*Lavandula augustifolia*
	Catnip	*Nepeta sibthorpii*
	Peppermint leaves	*Mentha piperita L.*
Withanolide	Indian ginseng, ashwagandha	*Withania somnifera*

The review also focuses on targets for inflammation, proliferation, apoptosis, invasion, metastasis and angiogenesis. Because a large portion of these nutraceuticals show great potential for targeting cancer through various mechanisms—such as the downregulation of transcription factors (e.g., nuclear factor-kappaB [NF-κB]), anti-apoptotic proteins (e.g., bcl-2, bcl-xL), promoters of cell proliferation (e.g., cyclooxygenase-2 [COX-2], cyclin D1, c-myc), invasive and metastatic genes (e.g., matrix metalloproteinases [MMPs], intracellular adhesion molecule-1 (ICAM-1), and angiogenic protein (vascular endothelial growth factor (VEGF)) (Table 2); and other uses of these triterpenoids are shown in Table 3. This review summarizes the sources and structures of triterpenoids and provides insight into the underlying molecular targets for cancer prevention and therapy.

**Table 2:** Molecular targets of triterpenoid for anticancer and anti-inflammatory activities.

Triterpenoid	Targets	References
Amyrin	NF-κB, IL-1β, COX-2, CREB, ERK, PKC, P38 MAPK	[9,10]
Avicin	NF-κB, Fas, STAT3, caspase-8, Bcl-2, Bcl-xL	[11,12,13,14,15,16]
Asiatic acid	NF-κB, caspases-2, -3, -8 and -9, PARP, Bcl-2	[17,18,19,20,21,22,23,24]
Astragaloside	NF-κB, VCAM-1	[25]
Betulinic acid	NF-κB, STAT3, Bax, Bcl-2, Bcl-xL, FAK	[26,27,28,29,30,31,32,33,34,35,36,37,38]
Boswellic acid	NF-κB, STAT3, AR, p21, DR5, caspase-3 and -8	[32,39,40,41,42,43,44,45,46,47,48]
Celastrol	NF-κB, IAP1, IAP2, Bcl-2, Bcl-xL, c-FLIP, COX-2, survivin, cyclin D1, MMP9, VEGF, iNOS, Hsp90, cdc37, VEGFR	[49,50,51,52,53]
Cucurbitacin	Cyclin B1, cyclin D1, Mcl-1, cdc25C, STAT3, p53	[54,55,56,57,58,59]
Diosgenin	NF-κB, survivin, XIAP, cyclin D1, cdk-2, cdk-4, mTOR, JNK, HMG-CoA reductase, p53, AIF, p21 ras, β-catenin	[60,61,62,63,64,65]
Escin	NF-κB, STAT3, JAK2, cyclin D1, Bcl-2, Bcl-xL, survivin, Mcl-1, VEGF, COX-2, MMP9	[66,67]
Ganoderic acid	NF-κB, AP-1, NFATc1, cdk4, uPA, MMP2, MMP9,	[68,69,70,71,72]
Ginsenosides	NF-κB, Bax, caspase-3, caspase-8, Bcl-2, IAP, XIAP, cyclin B1, cyclin D, cdk2, cdk4, VEGF, MAPK, IL-1β, TNF-α, ICAM-1, JNK	[73,74,75,76]
Glycyrrhizin	NF-κB, AP-1, TLR2, COX-2, IL-1α, TNF-α	[77,78,79,80,81,82,83]
Glycyrrhetinic acid	NF-κB, H-ras, Bax, cytochrome C, Bcl-2, Bcl-xL, Bak, caspase-3, PPARγ	[84,85,86]
Gypenoside	NF-κB, PPAR , VCAM-1, TF, iNOS, Ras	[87,88,89,90,91,92]
Lupeol	NF-κB, cFLIP, survivin, Bax, caspase-3, caspase-9	[93,94,95,96,97,98,99,100,101,102]
Madecassic acid	iNOS, COX-2, TNF-α, IL-1, IL-6	[103]
Momordin	NF-κB, AP-1, Bcl-2, Bax, caspase-3, PARP	[104,105]
Oleandrin	NF-κB, AP-1, Fas, ERK, Akt, FGF-1	[106,107,108,109,110]
Oleanolic acid	NF-κB, mTOR, caspases-3, -8, and -9, ICAM-1, VEGF, PARP, Akt	[111,112,113,114]
Platycodon D	NF-κB, Egr-1, caspase-3	[115,116]
Pristimerin	NF-κB, PARP-1, JNK, Bax, p27, Bcl-2, Bcl-xL	[117,118,119,120]
Saikosaponins	NF-κB, NF-AT, AP-1, IL-6, TNF- , IFN- , PKC , JNK, p53, Fas/FasL	[121,122,123]
Ursolic acid	NF-κB, STAT3, Bcl-2, Bax, ICAM-1, p53, PKC	[114,124,125,126,127,128,129,130,131,132]
Withanolide	NF-κB, AP-1, IL-6, COX-2, Hsp70, Hsp90, Bax	[133,134,135,136,137,138]

AIF, apoptosis inducing factor; AMPK, 5' AMP-activated protein kinase; AP-1, activator protein-1; Apaf1, apoptotic protease activating factor 1; AR, androgen receptor; Bax, BCL2-associated X protein; Bfl-1/A1, BCL2-related protein A1; cdc, cell division cycle; cdk, cyclin-dependent kinase; cFLIP, cellular FLICE inhibitory protein; COX-2, cyclooxygenase-2; CREB, cAMP response element binding protein; DR, death receptor; EGFR, epidermal growth factor receptor; Egr-1, earyl growth response factor-1; ERK, extracellular signal-regulated kinase; FAK, focal adhesion kinase; FasL, Fas-ligand; FGF-1, fibroblast growth factor-1; GSK3β, glycogen synthase kinase-3β; HMG-CoA, 3-hydroxy-3-methylglutaryl-coenzyme A; Hsp, heat shock protein; IAP, inhibitor of apoptosis protein; ICAM-1, intercellular adhesion molecule-1; IFN-γ, interferon-γ; IL‑1, interleukin-1; iNOS, inducible nitric oxide synthase; JNK, c-Jun N-terminal kinase; MAPK, mitogen–activated protein kinase; Mcl-1, myeloid cell leukemia-1; MCP, monocyte chemotactic protein; MEK, MAPK/ERK kinase, MIP-2, macrophage-inflammatory protein-2; MMP, matrix metalloproteinase; mTOR, mammalian target of rapamycin; NF-AT, nuclear factor of activated T-cells; NF–κB, nuclear factor–kappa B; PARP, poly (ADP-ribose) polymerase; PI3K, phosphoinositide-3 kinase; PKC, protein kinase C; PPAR, peroxisome proliferator–activated receptor; Sp1, specificity protein 1; STAT3, signal transducer and activator of transcription 3; TF, tissue factor; TLR2, Toll-like receptor-2; TNF-α, tumor necrosis factor-α; TRAF1, TNF receptor-associated factor-1; uPA, urokinase-type plasminogen activator; VCAM-1, vascular cell adhesion molecule-1; VEGF, vascular endothelial growth factor; VEGFR, VEGF receptor; XIAP, X-linked IAP.

**Table 3:** Other uses of triterpenoid in treatment of chronic diseases.

Disease	Triterpenoid
Diabetes	Astragaloside, Cucurbitacin, Diosgenin, Ginsenoside, Amyrin, Asiatic acid, Avicin, Betulinic acid, Escin, Glycyrrhizin, Oleanolic acid, Platycodon D, Ursolic acid, Withanolide
Cardiovascular	Astragaloside, Cucurbitacin, Diosgenin, Ginsenoside, Gypenoside, Oleandrin, Betulinic acid, Escin, Glycyrrhizin, Lupeol, Oleanolic acid, Platycodon D, Saikosaponins, Ursolic acid, Withanolide
Arthritis	Cucurbitacin, Diosgenin, Ginsenoside, Amyrin, Boswellic acid, Celastrol, Glycyrrhizin, Lupeol, Oleanolic acid, CDDO-Me, Ursolic acid, Withanolide,
Atherosclerosis	Diosgenin, Gypenoside, Betulinic acid, Glycyrrhizin, Oleanolic acid, Ursolic acid
Obesity	Diosgenin, Ginsenoside, Betulinic acid, Escin, Glycyrrhizin, Platycodon D, Momordin, Oleanolic acid, Ursolic acid
Alzheimer	CDDO-MA, Alpha-onocerin
Parkinson	CDDO-MA
Multiple sclerosis	Oleanolic acid
Depression	Asiatic acid
Osteoporosis	Ursolic acid
Cerebral ischemia	Escin, Asiatic acid
Memory loss	CDDO-MA

## 2. Source and Structure of Triterpenoids

**Figure 4:**
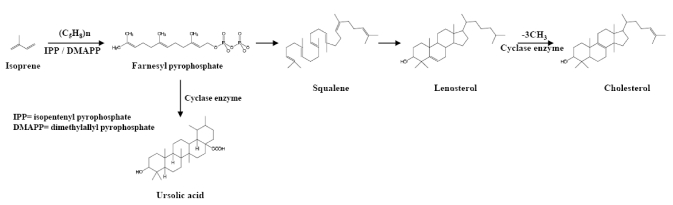
Different patterns of cyclization of squalene to form triterpenoids. (IPP, isopentenyl pyrophosphate; DMAPP, dimethylallyl pyrophosphate).

Triterpenoids are metabolites of isopentenyl pyrophosphate oligomers that are chemically related to squalene, which is a large group of compounds having 30 carbon atoms arranged in five rings with several oxygen atoms attached. Triterpenoids are part of the largest group of plant products, Saponins can be chemically biosynthesized when one or more sugar moieties attach to aglycone. There are two types of saponins, steroidal aglycone and triterpenoid aglycone. Both steroid and triterpenoid systems are found to be biosynthesized from a common precursor such as squalene [139]. Triterpenoids are synthesized from isopentenyl pyrophosphate (IPP) and its isomer dimethylallyl pyrophosphate (DMAPP). For this cyclization, three prenyltransferases synthesize the linear prenyl pyrophosphates geranyl pyrophosphate (GPP), farnesyl pyrophosphate (FPP), and geranylgeranyl pyrophosphate (GGPP). Squalene is in turn derived biosynthetically by the cyclization of a number of units of isoprene, (C_5_H_8_)n, which undergo folding through 20 different patterns in the presence of prenyl pyrophosphates to form monocyclic, dicyclic, tricyclic, tetracyclic, or pentacyclic derivatives [140]. A family of oxidosqualene cyclases may produce only a single product, such as lupeol cyclases, but there are also multifunctional oxidosqualene cyclases that use dammarenyl cation intermediates to produce many products. Once squalene undergoes cyclization, it goes through the cytosolic mevalonate pathway to make a proximate tetracyclic C30 compound, lanosterol (Figure 4), which further undergoes oxidation and catabolic metabolism to form cholesterol. 

The variety of triterpenoids in nature is a result of the evolution of a large terpene synthase superfamily. One study analyzed the amino acid sequences of terpene synthase genes and found that all originated from an ancestral diterpene synthase. It was also found that the diversity of these triterpenoids is due to the structural features of their catalyst enzymes. Terpenes and their metabolites are widely distributed in various plant systems that depend on various biotic and abiotic environmental factors. Terpenes and their metabolites are used in several developmental and physiological functions on the basis of the differential expression profiles of terpene synthase genes. Terpenes and their metabolites play a very important role in a plant’s defense mechanism. They protect the plants from both constitutive and induced defensive responses against insects and environmental stress [141,142]. Hence, triterpenoids provide a very good protection shield for plants, indicating their potential for use in the prevention of various cancers and inflammatory diseases in humans. 

## 3. Molecular Targets of Triterpenoids

In 1856, Rudolf Virchow for the first time showed inflammation to be a predisposing factor for various types of cancer. Today, the data suggest that at least one in seven malignant tumors diagnosed worldwide results from chronic inflammation and infection. Recognition of this fact has led to greater interest in research for molecular targets involved in the inflammatory pathways that trigger cancer and to find novel markers that restrain cancer progression along these pathways.

The conventional methods of treatment of cancer include surgery, chemotherapy and/or radiotherapy; the mode of treatment depends largely upon the type of cancer the patient has. Innovative, so-called multitargeting therapies from natural resources are urgently needed to target the various steps of cancer progression or the processes involved in cancer cell survival and metastasis to other parts of the body. 

It is clear now that cancer is not a simple disease involving a single gene, but a complex disease involving interaction between multiple genes, either within the same cell or with those of neighboring tissues. The prevention or progression of human cancer depends on the integrity of a complex network of defense mechanisms in which 300–500 genes have gone wrong, leading to the upregulation of undesired products such as antiapoptotic proteins or the downregulation of tumor suppressor proteins. 

### 3.1. NF-κB

NF-κB, a ubiquitous transcription factor, was discovered in 1986 as a nuclear factor that binds to the enhancer region of the κB chain of immunoglobulin in B cells. It is present in all cells, and in its resting stage, this factor resides in the cytoplasm as a heterotrimer consisting of p50, p65, and inhibitory subunit IκBα. NF-κB is activated by free radicals, inflammatory stimuli, cytokines, carcinogens, tumor promoters, endotoxins, γ-radiation, ultraviolet light, and x-rays [143]. On activation, the IκBα protein, an inhibitor of NF-κB, undergoes phosphorylation, ubiquitination, and degradation. p50 and p65 are then released to be translocated to the nucleus, bind to specific DNA sequences present in the promoters of various genes, and initiate the transcription of more than 400 genes. The kinase that causes the phosphorylation of IκBα is called IκBα kinase (IKK). Whereas the IKKβ mediates the classic/canonical NF-κB activation pathway, the IKKκ mediates the noncanonical pathway. IKK itself must be activated before it can activate IκBα. More than a dozen kinases have been described that can activate IKK, including protein kinase B (Akt), mitogen-activated protein/extracellular signal-regulated kinase kinase 1 (MEKK1), MEKK3, transforming growth factor (TGF)–activating kinase 1 (TAK1), NF-κB–activating kinase, NF-κB–inducing kinase, protein kinase C, and the double-stranded RNA-dependent protein kinase (PKR).

### 3.2. STAT3

Signal transducer and activator of transcription 3 (STAT3), one of the major molecular targets of triterpenoids, was first identified in 1994 as a DNA-binding factor that selectively binds to the IL‑6‑responsive element in the promoter. The activation of STAT3 is regulated by the phosphorylation of tyrosine 705 by receptor and nonreceptor protein tyrosine kinases, including epidermal growth factor receptor (EGFR) kinase [144], Src [145], Janus-activated kinases (JAK) [146,147], and extracellular signal-regulated kinase (ERK) [148]. The phosphorylation of STAT3 in the cytoplasm leads to its dimerization, translocation into the nucleus, and DNA binding, which results in the regulation of several genes involved in cell proliferation, differentiation, and apoptosis.

### 3.3. Other Pathways

A large body of evidence signifies the role of inflammation in cancer development through mediators such as reactive oxygen species (ROS), free radicals, and inflammatory cytokines like tumor necrosis factor-α (TNFα), lymphotoxins, and angiogenic factors. Also known to influence oncogenesis are signaling pathways that in normal cells are involved in tissue homeostasis, such as the NF-κB, prostaglandin/cyclooxygenase-2 (COX-2), and p53 pathways; the DNA repair machinery; and a family of the Toll-like receptor proteins.

Some of the most commonly known molecular targets of triterpenoids involved in the treatment and prevention of cancer have been targeted according to comprehensive knowledge of tumor growth and metastasis. This approach will maximize the effect of triterpenoids and minimize side effects by multitargeting the cells or processes that enable cancer to survive and spread in humans. 

## 4. Role of Triterpenoids in Cancer Prevention

### 4.1. Role of Triterpenoids in Inflammation

Inflammation is derived from the Latin word ‘*inflammare* or *inflammatio*’, which means, “to set on fire.” Inflammation is a basic defense mechanism in which the body reacts against infections, irritations, or other injuries. The four key features of inflammation are redness, heat, swelling, and pain. Inflammation stimulates the immune response at the site of injury or infection and is itself stimulated by increases in blood supply and vascular permeability, which allow more infiltration of plasma and leukocytes from the blood into injured tissues. This particular type of immune response is important because it helps the body to ward off pathogens and also to initiate the healing process in the damaged tissues. This reaction is classified as acute inflammation. Studies have shown that chronic inflammation is a progenitor of tumor progression and that many cancers have been found to arise from sites of infection, chronic irritation, and inflammation. Inflammation orchestrates the microenvironment around tumors and allows them to progress by fostering proliferation, survival, and migration [149]. The inflammatory cells and the network of signaling molecules provided by the inflammatory microenvironment are necessary for the malignant progression of transformed cells. 

Inflammation promotes tumor development through both nonimmune and immune. NF-κB is a central transcription factor mediating inflammatory and innate immune responses. NF-κB may be activated by various factors, including cytokines, microbial pathogens, and oxidative, genotoxic, physiological, or chemical stress factors. In addition to these, proinflammatory cytokines and chronic infections can play an important role in the stimulation of IKK activity, which leads to constitutive NF-κB activation [150,151,152]. The activation of NF-κB through IKKβ plays a major role in inflammation induced tumor promotion and progression. Various proinflammatory factors like TNFα and Toll-like receptor ligands such as lipopolysaccharide (LPS) normally activate these pathways [153]. This activation signals the transcription of various cancer-promoting genes such as antiapoptotic genes, proangiogenic genes, and proinvasion genes [154]. NF-κB DNA binding is thought to result in the activation of a number of genes that lead to inflammatory diseases like Alzheimer disease and arthritis in addition to cancer [143].

Along with NF-κB, factors such as TNF and interleukins (IL-1β, IL-6, and IL-8) also serve as connecting links between inflammation and cancer. TNF is released mainly from macrophages and regulates immune cells. Its dysregulation and overproduction lead to cancer and other diseases. TNF also plays a role in the activation of NF-κB by binding to a TNF receptor present on the cell surface that in turn triggers a pathway that leads to the activation of IKK [3]. Interleukins are a group of cytokines released in the body from numerous cells in response to various stimuli. While IL-1β plays an important role in the inflammatory response against infection by increasing the expression of endothelial adhesion factors, thus allowing infiltration of leukocytes at the site of infection, IL-6 is a proinflammatory cytokine released in response to trauma or tissue damage. IL-8, a member of the CXC chemokine family also known as CXCL8, can function as a mitogenic, angiogenic, and mutagenic factor promoting cancer progression [155].

Inflammatory cells and their regulators are found to facilitate angiogenesis and promote the growth, invasion, and metastasis of tumor cells [156]. Normal levels of some enzymes like inducible nitric oxide synthase (iNOS) and COX-2 play an essential role in the physiological protective response to tissue injury, but if expressed in excessive amounts, these inflammatory enzymes may cause carcinogenesis [149,157,158]. In tumor tissue, levels of prostaglandins (PGs) are often elevated. PGs are endogenous mediators of inflammation and are formed from arachidonic acid by constitutive COX-1 and inducible COX-2. Production of higher levels of PGs is thought to cause cellular injury and ultimately lead to carcinogenesis by inhibiting apoptosis, stimulating cellular proliferation, and promoting angiogenesis and tumor invasiveness [159]. Cycloartane triterpenoids from *Cimicifuga dahurica* suppressed the expression of cdc2 and COX-2 protein. These results imply that triterpenoids possess potential antitumor activities and exert their cytotoxicity through apoptosis and G2/M cell cycle arrest [146]. 

Many triterpenoids derived from botanical sources play an important role in reducing inflammation. These include avicin, asiatic acid, astragaloside, betulin, betulinic acid, boswellic acid, celastrol, cucurbitacin, diosgenin, erythrodiol, ganoderiol, ginsenosides, glycyrrhizin, glycyrrhetinic acid, gypenoside, lupeol, madecassic acid, maslinic acid, oleandrin, oleanolic acid, platycodon D, pristimerin, saikosaponins, ursolic acid, and withanolide (Table 2). Many of these triterpenoids target NF-κB, leading to its downregulation.

Pentacyclic triterpenoids have been found to have many functions, although their effective concentrations for various cellular effects may vary widely. Depending upon the dose administered, triterpenoids can induce anti-inflammatory, cytoprotective, tumor-differentiating, proliferation–arresting, and apoptotic effects [160,161,162]. The anticancer activities of triterpenoids appear to be mediated, at least in part, by their common ability to block TNF-induced NF-κB activation by inhibiting IKKβ [163,164]. The synthetic triterpenoid 1-[2-cyano-3,12-dioxooleana-1,9(11)-dien-28-oyl] imidazole (CDDO-Im) blocks NF-κB activation through direct inhibition of IKKβ [35]. This is evident from the fact that the molecular targets of the synthetic oleanane triterpenoids include IKK and also pathways involving STAT, IL-6, TGF‑β, and KEAP1 (the inhibitor of the transcription factor Nrf2). Inhibition of multiple targets by triterpenoids is believed to be mediated by the promiscuous reversible Michael addition of these compounds to exposed nucleophilic groups (such as accessible cysteine sulfides) of various susceptible signaling proteins [165]. Triterpenoids affect multiple signaling pathways, and the clinical properties of triterpenoids, particularly those of pentacyclic triterpenoids, have been shown in various studies. The structure-activity relationships indicate that the presence of α,β-unsaturated carbonyl moieties significantly enhance the potency of these pentacyclic triterpenoids. Of the 12 pentacyclic triterpenoids, four (CDDO, CDDO-Me, pristimerin, and celastrol) have been shown to be potently and selectively lethal to different cancer cells and show a several fold increase in anti-inflammatory activity. This action is caused by the α,β-unsaturated carbonyl in ring A. The incorporation of a cyano and keto group within this enone moiety further enhances its efficacy and potency.

Avicins are electrophilic pentacyclic triterpenoids with proapoptotic, anti-inflammatory, and antioxidant properties derived from *Acacia victoriae*. Avicins have been shown to induce redox‑dependent post-translational modification of cysteine residues to regulate protein function, which downregulate both STAT3 activity and the expression of STAT3-regulated prosurvival proteins and contribute to the induction of apoptosis *in vitro* [166]. Avicins were found to be potent inhibitors of TNF-induced NF-κB and to slow the accumulation of the p65 subunit of NF-κB in the nucleus; however, the degradation of IκBα was unaffected. In addition Avicins blocked the binding of NF-κB to DNA in *in vitro* binding assays. Treatment of cells with dithiothreitol (DTT) totally reversed the avicin G-induced inhibition of NF-κB activity, suggesting that sulfhydryl groups critical for NF-κB activation were being affected. Avicin G treatment decreased the expression of NF-κB regulated proteins such as iNOS and COX-2 [16]. Other studies showed that pretreating cells with triterpenoids for 24 hours significantly reduced the induction of NF-κB mediated through TNF-α [44,167]. 

Pristimerin, a natural triterpenoid, elicits cellular responses closely resembling those elicited by proteasome inhibitors, such as the rapid induction of heat shock proteins (HSPs), activating transcription factor 3 (ATF3), and C/EBP homologous protein (CHOP). Pristimerin also inhibits NF‑κB activation by inhibiting IKK-α or IKK-β, whereas proteasome inhibitors instead suppress NF‑κB function by impairing the degradation of ubiquitinated IκB. By inhibiting both IKK and the proteasome, pristimerin suppresses the activation of constitutive NF-κB in myeloma cells. Multiple myeloma is exquisitely sensitive to proteasome or NF-κB pathway inhibition. Consistent with this, pristimerin has been shown to be potently and selectively lethal to primary myeloma cells (IC_50_ < 100 nM) and to inhibit xenografted plasmacytoma tumors in mice [118]. Pristimerin is also known as an antifungal, antimicrobial, and anti-inflammatory plant compound with an effect on the iNOS system in LPS-activated RAW 264.7 macrophages [168]. 

Celastrol, a natural triterpenoid with a structure similar to that of pristimerin, is found in the thunder god vine and was identified as having potential for use in cancer treatment because of its ability to enhance the death of melanoma cells. Celastrol also inhibited cell proliferation in melanoma cells. When celastrol was used to treat melanoma cells, it (like pristimerin) increased levels of ubiquitinated proteins, reduced levels of TNF-α-induced IκB phosphorylation, and blocked NF-κB translocation to the nucleus at nanomolar concentrations; however, the molecular mechanism for these effects differed. Celastrol normally inhibits LPS-induced phosphorylation of mitogen-activated protein kinases/extracellular signal-regulated kinases 1/2 (MAPK/ERK1/2) and the DNA binding activity of NF-κB [49]. Other studies have indicated that TNF-induced IKK activation requires the activation of TAK1 and that celastrol inhibits the TAK1-induced NF-κB activation [52]. Celastrol also suppressed the ovalbumin-induced airway inflammation, hyperresponsiveness, and tissue remodeling by regulating the imbalance of matrix metalloproteinase 2 and 9 (MMP2, MMP9) and tissue inhibitor of metalloproteinase 1 and 2 (TIMP1 and TIMP2) by inflammatory cytokines through MAP kinases and NF-κB in inflammatory cells.

The triterpenoids erythrodiol and madecassic acid are structural analogues of each other and have antiproliferative and anticancer activity. However, only madecassic acid has also shown LPS‑stimulated NF-κB inhibition with subsequent blocking of p65 protein translocation to the nucleus [103]. This may be because of the presence of an additional hydroxyl at carbon 2, which play an important role in the electrophilic reaction. Maslinic acid, which is similar to madecassic acid, also inhibits NF-κB translocation. Maslinic acid also inhibited p50, p65, and NF-κB translocation in a dose-dependent manner in both unstimulated and phorbol-myristate acetate (PMA)-challenged cells, being particularly effective on the p50 subunit [169]. Momordin, an analogue of maslinic acid, does not contain any hydroxyl group at the carbon 2 position but still it has shown NF-κB inhibition in osteoclast differentiation. This may be because of momordin’s action on c-Fos, a component of the activating protein-1 (AP-1) transcription factor that plays a key role in osteoclast differentiation. Momordin inhibited the activation of NF-κB as well as AP-1 in receptor activator of NF-κB ligand (RANKL)-induced RAW264.7 cells, in which momordin appeared to target IκB degradation and c-Fos expression, but not MAPK signaling pathways [104]. 

Saikosaponins are triterpene saponins derived from the medicinal plant *Bupleurum falcatum* L. (Umbelliferae) that have shown various pharmacological and immunomodulatory activities including anti-inflammatory, antibacterial, antiviral and anticancer effects in ACHN, C32, Caco-2 , A375, A549, and Huh-7D12 cell lines [170]. Studies demonstrated that saikosaponins not only suppressed the proliferation of human T cells costimulated with OKT3 and CD28 but also inhibited PMA-, PMA/ionomycin-, and concanavalin A-induced mouse T-cell activation *in vitro*. This inhibitory effect of saikosaponins on PMA-induced T-cell activation was associated with the downregulation of NF-κB signaling through the suppression of IKK and Akt activities. Saikosaponins also suppressed both the DNA binding activity and the nuclear translocation of nuclear factor of activated T cells (NF-AT) and AP-1 in the PMA/ionomycin-stimulated T cells. In addition, the cell surface markers like IL-2 receptor (CD25) were also downregulated, and the production of proinflammatory cytokines such as IL-6, TNF-α, and interferon (IFN)-γ was decreased. These results indicate that the NF-κB, NF-AT, and AP‑1 (c-Fos) signaling pathways are involved in the T cell inhibition evoked by saikosaponins, demonstrating their potential for treating T cell-mediated autoimmune conditions [123]. Another study showed that saikosaponins have direct involvement in p53-, NF-κB- and Fas/Fas ligand-mediated induction of apoptosis and cell cycle arrest in human hepatoma cell lines. Saikosaponins also inhibited cell survival signaling by enhancing the amount of IκBα in the cytoplasm and reducing the level and activity of NF-κB in the nucleus, and subsequently attenuated the expression of bcl-xL in HepG2 and Hep3B cells. Saikosaponins therefore decreased cell proliferation and induced apoptosis both in p53‑positive HepG2 and p53-negative Hep3B cells [121]. 

Diosgenin, a steroidal triterpenoid having two pentacyclic rings, is present in *Trigonella foenum graecum* and other plants and has been shown to suppress inflammation, inhibit proliferation, and induce apoptosis in a variety of tumor cells. Diosgenin inhibits osteoclastogenesis, invasion, and proliferation through the downregulation of Akt, IKK activation, and NF-κB-regulated gene expression. Diosgenin suppresses NF-κB through direct DNA binding, activation of IKK, IκBα phosphorylation, IκBα degradation, p65 phosphorylation, and p65 nuclear translocation by inhibiting Akt activation. NF-κB-dependent reporter gene expression was also abrogated by diosgenin [64]. Similar activity was found in *Withania somnifera*, also known as Indian ginseng, which is widely used in the Ayurvedic system of medicine to treat tumors, inflammation, arthritis, asthma, and hypertension. Chemical investigation of the roots and leaves of this plant has yielded bioactive withanolides, a group of C28-steroidal lactone triterpenoids. *Withania somnifera* inhibits COX enzymes, lipid peroxidation, and proliferation of tumor cells, and it potentiates apoptosis, inhibits invasion, and abolishes osteoclastogenesis through the suppression of NF-κB activation and NF-κB-regulated gene expression [135]. 

Ursolic acid is a pentacyclic triterpene compound isolated from many types of medicinal plants and widely present in the human diet. It has been reported to possess a wide range of pharmacological properties and is one of the most promising chemopreventive agents for cancer. It has been shown to suppress the expression of several genes associated with tumorigenesis. It suppressed NF-κB activation induced by various carcinogens including TNF, PMA, okadaic acid, hydrogen peroxide (H_2_O_2_), and cigarette smoke condensate. Ursolic acid inhibited DNA binding of NF-κB. Ursolic acid inhibited IκBα degradation, IκBα phosphorylation, IKK activation, p65 phosphorylation, p65 nuclear translocation, and NF-κB-dependent reporter gene expression. Ursolic acid also inhibited NF-κB-dependent reporter gene expression activated by TNF receptor (TNFR), TNFR-associated death domain (TRADD), TNFR-associated factor (TRAF), NF-κB-inducing kinase (NIK), IKK, and p65 [125]. CDDO and CDDO-Me, two potent oleanane triterpenoids having structures similar to ursolic acid, are currently in Phase I clinical trials for the treatment of leukemia and solid tumors. CDDO blocks the action of NF-κB by preventing the nuclear translocation of p65; this blocks the transactivation of the *NOS2* and *PTGS2* genes, thus playing an anti-inflammatory role and causing cell cycle arrest. Cucurbitacin combined with CDDO has been shown to bring about apoptosis by inhibiting NF-κB activation, IκBα phosphorylation and degradation, NF-κB-reporter gene expression induced by TNF, and STAT signaling. Some other triterpenoids like astragaloside, boswellic acids, celastrol, ganoderiol F, and gypenoside also blocked the action of NF-κB, inhibiting the transactivation of *cox-2* [44,51,171,172,173]. CDDO, at nanomolar concentrations, suppresses the *de novo* synthesis of the inflammatory enzymes iNOS and COX-2 in activated macrophages because they contain α,β‑unsaturated carbonyl moieties. Since iNOS and COX-2 overexpression have been implicated as possible enhancers of carcinogenesis, CDDO has potential to be used as a chemopreventive agent. Furthermore, CDDO may also serve as a chemotherapeutic agent, as micromolar to nanomolar concentrations effectively induced differentiation of human myeloid leukemia cells, inhibited the proliferation of various human tumor cell types, and induced apoptosis in human myeloid and lymphocytic leukemia cells, osteosarcoma cells, and breast cancer cells, including cell lines resistant to chemotherapy [160].

Boswellic acids, a type of pentacyclic triterpenoid, have been shown to induce apoptosis in different cancer cells. At the molecular level, these compounds inhibit constitutively activated NF-κB signaling by intercepting the IKK activity; signaling through the IFN-stimulated response element remained unaffected, suggesting specificity for IKK inhibition [45,46]. In a xenograph study of animal meningioma cells, boswellic acids were found to have potent cytotoxic activity with IC_50 _values in the range of 2–8 μM. At low micromolar concentrations, boswellic acids rapidly and potently inhibited the phosphorylation of ERK-1/2 and impaired the motility of meningioma cells stimulated with platelet derived growth factor (PDGF) BB. The cytotoxic action of boswellic acids on meningioma cells may be mediated, at least in part, by the inhibition of the ERK signal transduction pathway, which plays an important role in signal transduction and tumorigenesis [174].

Platycodon, a triterpenoid isolated from *Platycodon grandiflorum*, showed chemopreventive effects on tumor invasion and migration in HT-1080 tumor cells. Platycodon reduced PMA-enhanced MMP9 and MMP2 activation in a dose dependent manner. Platycodon suppressed PMA enhanced expression of MMP9 protein as well as mRNA and transcription activity levels through the suppression of NF-κB activation without changing the TIMP1 levels. Platycodon also reduced PMA-enhanced expression of MMP2 active forms through the suppression of membrane-type 1 MMP (MT1-MMP), but platycodon did not alter MMP2 and TIMP2 levels. Moreover, ROS production induced by PMA was partly decreased in the presence of platycodon, and this suppression of ROS production may be related to diminished NF-κB activity [175]. In this case, NF-κB inhibition is totally ROS mediated, and most of these ROS are released from glucose molecules that are present on the side chain. Platycodon has been shown to be cytotoxic and to inhibit telomerase activity by downregulating hTERT expression at concentrations between 10 and 20 μM. Platycodon also reduced c-Myc and SP1 protein levels and DNA binding activities in a dose-dependent manner [176] and suppressed the LPS-induced expression of iNOS and COX-2 genes by suppressing NF-κB activation at the transcriptional level [51]. Platycodon also enhanced the mRNA expression of cytokines IL-2, IFN-γ, IL-4, and IL-10 and transcription factors T‑bet and GATA-3 in mice splenocyte induced by concanavalin A. This suggests that the number of sugar residues in the glycosidic chains attached to C-3 of aglycone could affect the hemolytic and adjuvant activities of platycodigenin-type saponins [177]. Betulinic acid suppressed NF-κB-dependent reporter gene expression and the production of NF-κB-regulated gene products such as COX-2 and MMP9, which are induced by inflammatory stimuli. It also suppressed TNF-induced apoptosis through the activation of NF-κB and NF-κB-regulated gene expression induced by carcinogens and inflammatory stimuli [35].

### 4.2. Role of Triterpenoids in Tumor Cell Survival, Apoptosis, and Proliferation

Apoptosis, which in Greek literally means “falling away,” is a process of programmed cell death that occurs normally in multicellular organisms. Apoptosis is a natural, organized process that plays an important role in embryonic development and adult tissue equilibrium by adjusting the physiological processes involved. The human body is made up of six trillion cells, with approximately three billion cells replaced every minute. Through the process of apoptosis, the body can eliminate damaged or unneeded cells without local inflammation from the leakage of cell contents [178]. 

Because deregulation of apoptosis is one of the most important factors involved in tumor cell progression, a number of scientific studies have been done on this process to determine if it can be exploited in cancer treatment. Apoptosis is the human body’s mechanism for destroying any cell that has abnormalities such as DNA damage, oncogene activation, nutrient deficiency, or hypoxia. But cancer cells have the ability to escape apoptosis, allowing tumors to grow rapidly and uncontrollably. 

Apoptosis in cancer cells can be triggered by the activation of proteases such as caspases, leading to the cells’ destruction. There are two different pathways by which this apoptosis can be stimulated in cancer cells. The first is the intrinsic pathway through mitochondria, which releases cytochrome C proteins such as second mitochondria derived activator of caspases (SMACs) that bind to and deactivate inhibitor of apoptosis proteins (IAPs), allowing apoptosis to proceed. Apoptotic signals in this pathway may come in the form of members of the Bcl-2 family of proteins such as pro-apoptotic Bax, which can be upregulated by tumor suppressor protein p53 in response to DNA damage [179]. The second pathway is the extrinsic pathway, in which apoptosis is triggered by the activation of proapoptotic receptors such as death receptors 4 and 5 (DR4 and DR5) and Fas, which are present on the cell surface. The activation of the death receptor pathway leads to receptor aggregation, which then initiates the recruitment and activation of initiator caspase-8. While p53 is involved in the intrinsic pathway, it has no role in the extrinsic pathway [178,180,181,182].

STAT3 activation has been associated with cell survival, proliferation, and invasion in various human cancers. Some members of the Bcl-2 family of proteins, such as Bcl-2 and Bcl-xL, also play a role in apoptosis and have been found to be elevated in different types of cancer cells. These proteins cause some cells to develop resistance to drugs used in cancer treatment. Another protein, survivin, may play a role in tumor progression as it has been found at excessive levels in cancer cells. Targets for the treatment of cancer could be those that cause downregulation of the Bcl-2, Bcl-xL, and survivin proteins and upregulation of the p53, Bax, and caspase proteins.

Triterpenoids have been found to act through the intrinsic apoptosis pathway to prevent tumor progression. For example, many spice derived triterpenoids have been shown to induce apoptosis in different types of cancer cells through a wide variety of mechanisms. Among the most important of these are asiatic acid, astragaloside, celastrol, cucurbitacin, diosgenin, gypenoside, hederagenin, lupeol, and momordin (Table 2). These triterpenoids have a common target, the antiapoptotic protein Bcl-2, which can induce apoptosis in cancer cells.

Pristimerin has been shown to induce mitochondrial cell death in human cancer cells, and the ROS dependent activation of both Bax and poly (ADP-ribose) polymerase-1 (PARP-1) is critically required for mitochondrial dysfunction [117,183]. In human HL-60 cells, pristimerin also showed antiproliferative effects, with an IC_50_ of 0.88 μM [184]. In addition to this, pristimerin showed that c‑Jun N-terminal kinase (JNK) was involved in ROS-dependent Bax activation, which increases intracellular ROS, JNK activation, conformational change, and mitochondrial redistribution of Bax, mitochondrial membrane potential loss, and cell death. Pretreatment with pristimerin also activated PARP-1 [117]. Another study showed that pristimerin induced apoptosis by targeting the proteasome in prostate cancer cells. This may be because of the accumulation of ubiquitinated proteins and three proteasome target proteins, Bax, p27 and IκBα, in androgen receptor (AR)-negative PC-3 prostate cancer cells, which supports the conclusion that proteasome is inhibited by pristimerin [120]. Another study showed that this apoptosis might be induced by pristimerin through the direct effect of caspase on mitochondria in MDA-MB-231 cells [119]. Pristimerin showed antiviral activity by inhibiting the viral DNA synthesis but had no virucidal effect [185]. 

Celastrol combined with TNF-related apoptosis-inducing ligand (TRAIL/APO-2L) exerted strong synergistic antiproliferative effect against human cancer cells, including those from ovary cancer (OVCAR-8), colon cancer (SW620), and lung cancer (95-D). *In vivo*, the antitumor efficacy of TRAIL/APO-2L was dramatically increased by celastrol. These enhanced anticancer activities were accompanied by the prompt onset of caspase-mediated apoptosis. Celastrol also suppressed the TNF‑induced expression of various gene products involved in antiapoptosis (IAP1, IAP2, Bcl-2, Bcl‑xL, c-FLIP, and survivin), proliferation (cyclin D1 and COX-2), invasion (MMP9), and angiogenesis (VEGF) [52]. 

Diosgenin-induced apoptosis was associated with COX-2 upregulation in HEL cells [75]. Diosgenin also downregulated gene products involved in cell proliferation (cyclin D1, COX-2, and c‑myc) and antiapoptosis (IAP1, Bcl-2, Bcl-xL, Bfl-1/A1, TRAF1, and cFLIP) [64].

Avicins are novel plant derived metabolites that lower energy metabolism in tumor cells by targeting the outer mitochondrial membrane Avicins dephosphorylated STAT3 in a variety of human tumor cell lines, leading to a decrease in the transcriptional activity of STAT3. The expression of STAT3-regulated proteins such as c-myc, cyclin D1, Bcl-2, survivin, and VEGF were reduced in response to avicin treatment. Avicin also induced dephosphorylation of STAT3, dephosphorylation of JAKs, and activation of protein phosphatase-1 [11]. Another study showed that avicins induced apoptosis and downregulated p-STAT3, Bcl-2, and survivin in cutaneous T-cell lymphoma cells. Avicin D did not change STAT3 expression, but it decreased phospho-STAT3 protein levels [13,187]. 

Betulinic acid inhibited the constitutive activation of STAT3, Src kinase, JAK1, and JAK2. Pervanadate reversed the betulinic acid induced down regulation of STAT3 activation, suggesting the involvement of a protein tyrosine phosphatase (PTP). Betulinic acid also downregulated the expression of STAT3 regulated gene products, such as Bcl-xL, Bcl-2, cyclin D1, and survivin. This correlated with an increase in apoptosis as indicated by an increase in the sub-G1 cell population and an increase in caspase-3-induced PARP cleavage [188]. 

Recently, some researchers found that natural triterpenic diols promote apoptosis in astrocytoma cells through ROS-mediated mitochondrial depolarization and JNK activation. Alcohols extracted from olive oil, erythrodiol (an intermediate from oleanolic acid), and its isomer, uvaol, have been reported anticancerous, particularly on brain cancer cells. Erythrodiol and uvaol effectively affected cell proliferation as well as cell cycle phases and induced 1321N1 cell death and modulated the apoptotic response, promoting nuclear condensation and fragmentation. These results may be due to production of ROS with loss of mitochondrial transmembrane potential, and correlated with the activation of JNK. The presence of catalase reversed the triterpenic diols induced mitochondrial depolarization, JNK activation, and apoptotic death, indicating the critical role of ROS in the action of diols ring compounds [189]. Oleanolic acid also upregulated COX-2 expression and induced prostacyclin (PGI2) synthesis. These effects may be as a result of the early activation of cAMP regulatory element-binding protein (CREB), a key transcription factor involved in COX-2 transcriptional upregulation. Oleanolic acid has also shown cardioprotective effects [190].

Maslinic acid is present in high concentrations in olive pomace. Various studies have examined the responses of HT-29 and Caco-2 colon cancer cell lines to maslinic acid treatment. It also induced strong G0/G1 cell-cycle arrest and DNA fragmentation, and increased caspase-3 activity. However, maslinic acid did not alter the cell cycle or induce apoptosis in the non-tumorous intestinal cell lines IEC-6 and IEC-18 [191]. 

Momordin inhibited proliferation and induced apoptosis in human promyelocytic leukemia (HL-60) cells and was cytotoxic to HL-60 cells with an IC_50_ of 19.0 μg/mL. The antiproliferative effects of momordin appear to be attributable to its induction of apoptotic cell death, as momordin induced nuclear morphology changes and internucleosomal DNA fragmentation and increased the proportion of hypodiploid cells. Momordin decreased the expression of the antiapoptotic protein Bcl-2 but increased the expression of the proapoptotic protein Bax. In addition, treatment with momordin induced the activation of caspase-3 and the cleavage of PARP [105]. Many of the triterpenoids derived from nature, target caspases, which are essential for apoptotic cell death.

Saikosaponins were found to be cytotoxic in different cancer cell lines and to exert significant inhibition of nitric oxide production in LPS-induced RAW 264.7 macrophages, with IC_50_ of 4.2 and 10.4 μM, respectively [170]. Saikosaponins have shown a variety of pharmacological and immunomodulatory activities, including anti-inflammatory, antibacterial, antiviral and anticancer effects. Treatment of MDA-MB-231 with saikosaponin-A increased the population of cells in the sub‑G1 phases of the cell cycle. These results showed that apoptosis in MDA-MB-231 cells was independent of the p53/p21 pathway mechanism and was accompanied by an increased ratio of Bax to Bcl-2, increased c-myc levels, and increased activation of caspase-3. In contrast, apoptosis of MCF7 cells may have been initiated by the Bcl-2 family of proteins and involved the p53/p21-dependent pathway mechanism, and it was accompanied by an increased level of c-myc protein [192]. In another study, an enhancement in Fas and its two ligands, membrane-bound Fas ligand (mFasL) and soluble Fas ligand (sFasL), as well as Bax protein, was shown to be responsible for the apoptotic effect induced by saikosaponins [121]. Saikosaponins significantly increased the levels of c-myc and p53 mRNA [193]. Saikosaponins also caused G0/G1 cell cycle arrest of activated T cells by downregulating the protein levels of CDK6 and cyclin D3 and upregulating the protein level of p27(kip) [194].

The inhibition of NF-κB activation by ursolic acid correlated with the suppression of NF-κB dependent cyclin D1, COX-2, and MMP9 expression [125]. Ursolic acid blocked cell cycle progression in the G1 phase and was associated with a marked decrease in the protein expression of cyclin D1, D2, and E, and their activating partners cdk2, cdk4, and cdk6 with concomitant induction of p21. The accumulation of p21/WAF1 might be p53 dependent. The accumulation of p21/WAF1 correlated with the upregulation of Fas, the Fas ligands and Bax, and the downregulation of NF-κB, Bcl-2, and Bcl-xL [121]. Ursolic acid also upregulated apoptotic genes *p53* and *caspase-3*, while the antiapoptotic gene *Bcl-2* was downregulated [124]. 

CDDO, concentrated at 1–5 μM, induced apoptosis in various cancer cell lines. Moreover, CDDO combined with TRAIL promoted the induction of apoptosis. CDDO normally acts through both the extrinsic and intrinsic pathways by activating the cleavage of BID and of caspases-3, -8, and -9; by downregulating FLIP; or by inducing the translocation of Bax to the mitochondria and the release of cytochrome C [195,196,197]. The antitumor activity of CDDO-Me was associated with the inhibition of p-Akt, mammalian target of rapamycin (mTOR), and NF-κB signaling proteins and their downstream targets such as p-Bad and p-Foxo3a for Akt; p-S6K1, p-eIF-4E and p-4E-BP1 for mTOR; and COX-2, VEGF and cyclin D1 for NF-κB [167].

Acetyl-11-keto-β-boswellic acid (AKBA), a derivative of boswellic acid has been shown to induce apoptosis in cancer cells. AKBA mediated inhibition of the phosphatidylinositol-3-kinase (PI3K)/Akt pathway; this pathway is crucial for cell proliferation and survival [198]. Another study showed that cyclin D1 and E, CDK2 and 4 and phosphorylated retinoblastoma protein (Rb) were decreased in AKBA-treated cells, while p21expression was increased. The growth inhibitory effect of AKBA was dependent on p21 but not p53 [199]. The cytostatic and apoptosis inducing activities of boswellic acids toward malignant cell lines has been shown *in vitro* [200]. Boswellic acids triggered apoptosis by means of a pathway dependent on caspase-8 activation but independent of Fas/Fas ligand interaction in colon cancer cells [201]. AKBA inhibited the NF-κB-dependent reporter gene expression activated by TNFR, TRADD, TRAF2, NIK, and IKK, but not that activated by the p65 subunit of NF-κB, which indicates that AKBA enhances apoptosis induced by cytokines and chemotherapeutic agents [46].

In HT-29 human colon cancer cells, platycodon induced apoptosis through DNA fragmentation and PARP cleavage. The apoptosis induced by platycodon was associated with the activation of initiator caspases-8 and -9 as well as effector caspase-3. Platycodon stimulated Bid cleavage, indicating that the apoptotic action of caspase-8-mediated Bid cleavage leads to the activation of caspase-9. It increased the expression of the proapoptotic protein Bax and decreased the expression of the antiapoptotic protein Bcl-2. Platycodon also increased the expression of the caspase-independent mitochondrial apoptosis factor, apoptosis inducing factor (AIF), in HT-29 cells. Thus, platycodon exerts its apoptotic effect via both caspase-dependent and caspase-independent pathways [116,202,203].

A few triterpenoid compounds have been shown to both activate caspase activity and downregulate the expression of Bcl-2 or Bcl-xL. The ability to suppress proliferation and induce apoptosis in human cancer cells is clearly important for drugs targeting either malignant cells in treatment or premalignant cells in prevention.

One of the hallmarks of cancer is aggressive proliferation of cells. In a normal cell, a fine balance between growth signals and antigrowth signals regulates proliferation. However, this fine orchestration is lost in cancer cells, which often show uncontrolled growth due to the loss of both growth-controlling factors. On one hand, cancer cells acquire the capability to generate their own growth signals, while on the other hand, they also become unresponsive to antigrowth signals [204]. Numerous factors regulate the natural progression of a normal cell. Some of these factors, such as cyclins, are upregulated in cancer cells, causing the cells to replicate uncontrollably. Cyclins are the regulatory proteins that control the cell cycle, while other factors such as COX-2 and c-myc play a supporting role. The most commonly affected cyclin in cancer cells is cyclin D1, an important cell cycle regulator that plays a role in transition of the cell from the G1 phase to the S phase. Cancer cells show overexpression of this cyclin D1 and thus it has been linked to the development and progression of cancer.

Avicins downregulate both STAT3 and the expression of STAT3-regulated prosurvival proteins, which contribute to the induction of apoptosis in tumor cells. STAT3 plays an important role in inflammation and wounding, and the *in vivo* inhibition of VEGF. In a mouse skin carcinogenesis model, avicins inhibited the expression of STAT3, resulting in the suppression of the pro-inflammatory and pro-oxidant stromal environment of tumors [11]. Avicins at concentrations of 0.5–5.0 μg/mL caused more apoptosis in patients' Sézary cells than in healthy donors' CD4+ T cells and activated CD4+ T cells and decreased apoptosis inhibitors bcl-2 and survivin [13]. Furthermore, avicin D-induced autophagic cell death was abrogated by knockdown of tuberous sclerosis complex 2 (TSC2), a key mediator linking AMP-activated protein kinase (AMPK) to mTOR inhibition, suggesting that AMPK activation is a crucial event targeted by avicins. Avicins also have been shown to lower energy of metabolism in tumor cells by targeting the outer mitochondrial membrane, causing cancer cell death [187]. Tumor cells generate hydroperoxides at a very high rate, and avicins could provide a new strategy of anticancer therapy by sensitizing cells with high levels of ROS to apoptosis. In another study, boswellic acids, which inhibit STAT3 activation, led to the suppression of gene products involved in proliferation (cyclin D1), survival (Bcl-2, Bcl-xL, and Mcl-1), and angiogenesis (VEGF) [32].

Maslinic acid has shown an antiproliferative effect against Caco-2 cancer cells (EC_50_ = 15 µM), HT-29 human colon cancer cells (EC_50_ = 74 µM), 1321N1 astrocytoma cells (EC_50_ = 25 µM), and human leukemia cells (CCRF-CEM and CEM/ADR5000) (EC_50_ = 7 and 9 µM, respectively). Maslinic acid’s antiproliferative activity likely comes from the induction of an oxidative apoptotic pathway, which causes cell cycle and cytoskeleton alterations. Maslinic acid has been found to attenuate intracellular oxidative stress by inhibiting of NO and H_2_O_2_ production and reducing proinflammatory cytokine generation in murine macrophages [205]. Maslinic acid inhibited cell growth with an EC_50_ of 101.2 μM without necrotic effects. This effect of maslinic acid is caused by a hydroxyl group at the carbon 2 position, ultimately activates caspase-3 as a prime apoptosis protease [206]. A 200 μM concentration of maslinic acid was sufficient for activating caspase-3, which inhibits cell proliferation [207]. Maslinic acid from pomace olive oil demonstrated a suppressive effect on oxidative stress and cytokine production in stimulated murine macrophages [208]. Triterpenoids isolated from apple peels have shown potent antiproliferative activity and may be partially responsible for apples’ anticancer activity.

Saikosaponins prevented the proliferation of MCF-7 cell at the concentration of 10 nM to 10 μM and was significantly inhibited by the specific estrogen receptor (ER) antagonist ICI-182780 [209]. This antiproliferative effect is due to the synthesis of extracellular matrix proteins through the downregulation of the *CDK4*, *c-Jun*, and *c-Fos* genes [210], which block cell cycle progression at the G1 phase.

The potency of ursolic acid was associated with ZIP/p62 and protein kinase C-zeta (PKC-zeta). Ursolic acid inhibited the interaction of ZIP/p62 and PKC-zeta. It also further suppressed the activation of NF-κB and the downregulation of the MMP9 protein, which in turn contributed to ursolic acid’s inhibitory effects on IL-1β or TNF-induced C6 glioma cell invasion [127]. Ursolic acid showed the strongest inhibitory activity to urokinase (IC_50_ = 12 μM) and cathepsin B (IC_50_ = 10 μM), and as proteases are involved in tumor invasion and metastasis, this activity could be beneficial in cancer treatment [211]. CDDO-Me inhibited growth and induced apoptosis in PC-3 and C4-2 cells and was associated with the inhibition of the p-Akt, mTOR, and NF-κB signaling proteins and COX-2, VEGF and cyclin D1 [167]. Some of the triterpenoid derivatives obtained from ursolic acid have shown ability to suppress the *de novo* formation of two enzymes, iNOS and COX-2, in IFN-γ-stimulated primary mouse macrophages or LPS-activated RAW 264.7 macrophages that are used as assay systems [212]. 

The inhibition of STAT3 activation by boswellic acids led to the suppression of gene products involved in proliferation (cyclin D1), survival (Bcl-2, Bcl-xL, and Mcl-1), and angiogenesis (VEGF. Betulinic acid combined with vincristine showed a synergistic cytotoxic effect on melanoma cells, inducing cell cycle arrest at different points (betulinic acid at G1 phase and vincristine at G2/M phase) and causing apoptosis in B16F10 melanoma cells. In C57BL/6 mice, vincristine inhibited metastasis of melanoma cells to the lung, an effect that was augmented by the addition of betulinic acid [213].

### 4.3. Role of Triterpenoids in Invasion, Metastasis, and Angiogenesis

Besides uncontrolled proliferation, the other major characteristics of cancer cells are invasion and metastasis. In metastasis the cancer cells migrate from their original site of origin to other parts of the body, either via the bloodstream or lymphatic system. Among the factors influencing invasion, which affects whether or not a tumor will metastasize, are MMPs and ICAM-1. MMPs (specifically MMP2 and MMP9) are endopeptidases that degrade the basement membrane components, separating the cells from their surrounding tissue and enabling them to move freely and spread to other tissues [214]. Chemokine receptor CCR7 is important for lymphatic invasion of cancer cells and is overexpressed in metastatic breast cancer cells; withanolide inhibits TAK1 to repress NF-κB-induced CCR7 expression in breast cancer cells and is useful for the prevention of lymphatic involvement by breast cancer cells [215].

Erythrodiol-3-acetate, a triterpenoid, reduced the level of MMP1 and induced type 1 procollagen in a dose dependent manner [216]. *Ganoderma lucidum*, a well known mushroom containing platycodon, showed a significant inhibitory effect on PMA-induced MMP9 and MMP2 activation in a dose dependent manner and further inhibited HT-1080 and HepG2 cell invasion and migration [217]. Another study found that boswellic acids potentiated the apoptosis induced by TNF and chemotherapeutic agents, suppressed TNF-induced invasion, and inhibited NF-κB-induced osteoclastogenesis [46].

Angiogenesis is the basis for solid tumor development and distribution, and antiangiogenic drugs have been demonstrated to be active at the site of cancer. The growth of human tumors and development of metastases depends on the *de novo* formation of blood vessels [218]. Angiogenesis, the physiological process in which new blood vessels develop from pre-existing ones, normally occurs during growth, reproduction, and wound healing; however, this process is also a marker indicating that a tumor has progressed from a dormant to malignant state. Angiogenesis favors tumor growth by providing oxygen and nutrients to multiplying cells via the newly formed blood vessels. Some proangiogenic factors that favor development of new blood vessels include IL-8, TNF, fibroblast growth factor-2 (FGF-2), and PDGF. However, the most important factor in the whole process of angiogenesis is VEGF. Various studies have shown it to be a potent stimulator of angiogenesis *in vitro* [218]. Because of its critical role in angiogenesis, it has been targeted for controlling tumor progression. Limiting VEGF in tumors has been shown to lead to blood vessel destruction and to prevent the growth of new ones, thus reducing the blood supply to the tumor. Inhibition of the VEGF tyrosine kinase signaling pathway blocked angiogenesis in growing tumors, leading to stasis and regression of the tumors. Thus, agents that can downregulate or inhibit the expression of VEGF or its signaling pathway in tumor cells could prove to be very promising in preventing tumor growth and metastasis.

Saikosaponin C, one of the saikosaponins present in a Chinese herb, *Radix bupleuri*, has been found to have a potent inducing effect on human umbilical vein endothelial cells’ viability and growth. Saikosaponin C also induced endothelial cell migration and capillary tube formation. Saikosaponin C induced the gene expression or activation of MMP2, VEGF, and the p42/p44 MAPK that correlates with endothelial cell growth, migration, and angiogenesis, respectively [219]. Another study found that saikosaponins can inhibit the physiological angiogenesis of chicken embryos, especially for the medium and small vessels [220].

CDDO-Me and CDDO-Im were shown to inhibit the activation of the ERK1/2 pathway after stimulation with VEGF in human umbilical vein endothelial cells [221]. CDDO-Me also potentiated the cytotoxic effects of TNF and chemotherapeutic agents. This may be because CDDO-Me inhibits NF-κB through the inhibition of IκBα kinase, leading to the suppression of NF-κB-regulated gene product (VEGF, COX-2, and MMP9) expression and to angiogenesis [222]. Boswellic acids suppressed VEGF-induced phosphorylation of VEGF receptor 2 (VEGFR2) kinase (KDR/Flk-1) with an IC_50_ of 1.68 μM. Specifically, boswellic acids suppressed the downstream protein kinases of VEGFR2, including Src family kinase, focal adhesion kinase, ERK, AKT, mTOR, and ribosomal protein S6 kinase [223]. In an *ex vivo* model, boswellic acids significantly inhibited blood vessel formation in the Matrigel plug assay in mice and effectively suppressed VEGF induced microvessel sprouting in a rat aortic ring assay. Furthermore, boswellic acids inhibited VEGF induced cell proliferation, chemotactic motility, and the formation of capillary-like structures from primary cultured human umbilical vascular endothelial cells in a dose-dependent manner [223]. Betulinic acid also inhibits growth factor-induced *in vitro* angiogenesis by modulating mitochondrial function in endothelial cells [224].

Various *in vivo* studies have found that celastrol can downregulate the density of tumor microvessels significantly at different doses. Immunohistochemistry showed that celastrol also decreased the levels of VEGFR1 and VEGFR2 expression, but not the level of VEGF expression [225]. However, avicins, downregulate the expression of VEGF [166]. An *in vivo* study showed that *Ganoderma lucidum* given at 100 and 200 mg/kg inhibited primary solid tumor growth in the spleen, liver metastasis, and secondary metastatic tumor growth in the liver in intrasplenic Lewis lung carcinoma-implanted mice. An *in vivo* assay system extract inhibited Matrigel induced angiogenesis [226]. 

## 5. Role of Triterpenoids in Cancer Treatment

Triterpenoids are structurally diverse organic compounds. More than 20,000 triterpenoid varieties are formed by multiple modifications of the basic backbone structure. Several triterpenoids such as avicin, betulinic acid, boswellic acids, celastrol, diosgenin, madecassic acid, maslinic acid, momordin, saikosaponins, platycodon, pristimerin, ursolic acid, CDDO, and withanolide, have been shown in our laboratory and others to possess anticancer and anti-inflammatory activities. Preliminary data from ongoing studies indicate that some synthetic triterpenoids are being developed with improved anticancer activity. Several triterpenoids are now in clinical trials at different phases (Table 4; www.clinicaltrials.gov). Synthetic triterpenoid, CDDO, is being tested in patients with various cancers. Betulinic acid ointment is under evaluation for the treatment of dysplastic nevi that have the potential to transform into melanoma. Triterpenoids are highly multifunctional and thus have promise as agents in the treatment of cancer because of their ability to block the NF-κB activation, induce apoptosis, and inhibit proliferation, invasion, metastasis and angiogenesis.

**Table 4:** List of triterpenoids in clinical trials.

Triterpenoids	Cancer	Phase	Status	Sponsors
CDDO-Me	Solid tumors or	I	Terminated	MDACC
	Lymphoid malignancies			
CDDO	Solid Tumors	I	Completed	NCI
	or Lymphoma			
CDDO-Me	Liver disease	I/II	Terminated	RPI
Ginsenoside	Breast cancer	II	Ongoing	SIU
Ginsenoside	Hypertension	II	Completed	SMH
Ginsenoside	Ischemic Stroke	II/III	Completed	XH
Betulinic acid	Dysplastic nervus syndrome	I/II	Ongoing	UI
Escin	Arm lymphedema	II	Completed	UW
Glycyrrhizin	Hepatitis C	III	Ongoing	SP
Glycyrrhetinic acid	End stage renal disease	II	Ongoing	UHI
Glycyrrhetinic acid	AME	II/III	Completed	BWH

MDACC, MD Anderson Cancer Center, U.S.; NCI, National Cancer Institute, U.S.; RPI, Reata Pharmaceuticals, Inc; SIU, Southern Illinois University; SMH, St. Michael's Hospital, Toronto; XH, Xijing Hospital, China; UI, University of Illinois; UW, University of Wisconsin, U.S.; SP, Schering-Plough; UHI, University Hospital Inselspital, Switzerland; AME, Apparent Mineralocorticoid Excess; BWH, Brigham and Women's Hospital, U.S.

## 6. Conclusions

This review has illustrated that triterpenoids are important active constituents obtained from various plants and could be considered for use in both the chemoprevention and chemotherapy of cancer. Inflammatory proteins and their pathways are critical targets in both the prevention and treatment of cancer. Therefore, identification of agents or drugs that can suppress these pathways is of enormous importance. 

Polycyclic triterpenoids now offer important new platforms for drug development. The natural triterpenoid platform provided by the unique stereochemistry of these particular compounds, and the cyclization of the linear 30-carbon squalene molecule, have provided an excellent base from which new agents more potent than the parent squalene could be developed. 

Thus, agents that can suppress NF-κB and activate caspase and other pathways (e.g., DR4, DR5) are likely to be effective drugs. Because of their safety and ability to affect multiple targets, natural products are likely to have a special place in the preventive and therapeutic armamentarium against cancer. Although there are extensive preclinical data to support such claims, only clinical studies can fully validate them. 
